# 546. Therapeutic Effect of Regdanvimab in Patients with Mild to Moderate COVID-19: Day 28 Results from a Multicentre, Randomised, Controlled Pivotal Trial

**DOI:** 10.1093/ofid/ofab466.745

**Published:** 2021-12-04

**Authors:** Michael G Ison, Jin Yong Kim, Oana Sandulescu, Liliana-Lucia Preotescu, Norma Erendira Rivera Martinez, Marta Dobryanska, Victoria Birlutiu, Egidia Gabriela Miftode, Natalia Gaibu, Olga Adriana Caliman-Sturdza, Simin-Aysel Florescu, Anca Streinu-Cercel, Sang Joon Lee, Sung Hyun Kim, Il Sung Chang, Yun Ju Bae, Jee Hye Suh, Mi Rim Kim, Da Re Chung, Sun Jung Kim, Seul Gi Lee, Ga Hee Park, Joong Sik Eom

**Affiliations:** 1 Northwestern University, Chicago, IL; 2 Division of Infectious Diseases, Department of Internal Medicine, Incheon Medical Center, Incheon, Inch'on-jikhalsi, Republic of Korea; 3 “Prof. Dr. Matei Bals” National Institute for Infectious Diseases, Carol Davila University of Medicine and Pharmacy, Bucharest, Bucuresti, Romania; 4 "Prof. Dr. Matei Bals" National Institute for Infectious Diseases, Bucharest, Bucuresti, Romania; 5 Oaxaca Site management Organization (OSMO) - PPDS, Oaxaca, Mexico; 6 City Clinical Hospital 12, Kyiv, Ukraine; 7 Lucian Blaga University of Sibiu, Faculty of Medicine. County Clinical Emergency Hospital, Sibiu, Romania; 8 Spitalul Clinic de Boli Infectioase “Sfanta Parascheva”, Iasi, Romania; 9 IMSP Republican Clinical Hospital, Chisinau, Moldova; 10 Stefan cel Mare University of Suceava, Sf. Ioan cel Nou Emergency County Hospital Suceava, Suceava, Romania; 11 Dr. Victor Babes Clinical Hospital For Tropical and Infectious Diseases, București, Romania; 12 Celltrion, Inc., Incheon, Inch'on-jikhalsi, Republic of Korea; 13 Gachon University Gil Medical Center, Incheon, Inch'on-jikhalsi, Republic of Korea

## Abstract

**Background:**

Regdanvimab is a monoclonal antibody with activity against SARS-CoV-2. A Phase 2/3 study with two parts is currently ongoing and data up to Day 28 of Part 1 is available while the data from 1315 patients enrolled in Part 2 are expected in June 2021.

**Methods:**

This phase 2/3, randomized, parallel-group, placebo-controlled, double-blind study with 2 parts is aimed to assess the therapeutic efficacy of regdanvimab in outpatients with mild to moderate COVID-19, not requiring supplemental oxygen therapy. Patients aged >18 with the onset of symptoms within 7 days were eligible to be enrolled.

**Results:**

In Part 1, 307 patients (101, 103, and 103 patients in the regdanvimab 40 mg/kg, regdanvimab 80 mg/kg, and placebo groups, respectively) were confirmed to have COIVD-19 by RT-qPCR at Day 1 (or Day 2). Regdanvimab significantly reduced the proportion of patients who required hospitalization or supplemental oxygen therapy compared to placebo (8.7% in the placebo vs. 4.0% in the regdanvimab 40 mg/kg). The difference in events rate was even larger in patients who met the high-risk criteria and confirmed a 66.1% reduction in patients receiving regdanvimab 40 mg/kg (Table 1). The median time to clinical recovery was shortened by 2.9 days (7.18 days for regdanvimab 40 mg/kg and 10.03 days for placebo; high-risk). Also, greater reductions from baseline viral load were shown in regdanvimab groups (Figure 1). The safety results confirmed that the regdanvimab was safe and well-tolerated. Occurrence of adverse events (Table 2) and results of other safety assessments were generally comparable among the 3 groups. The overall rate of infusion-related reaction was low and no serious adverse events or deaths were reported. The anti-drug antibody positive rate was low in the regdanvimab groups (1.4% in regdanvimab vs. 4.5% in placebo), and no antibody-dependent enhancement was reported.

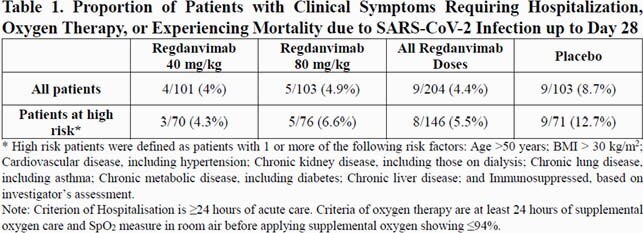

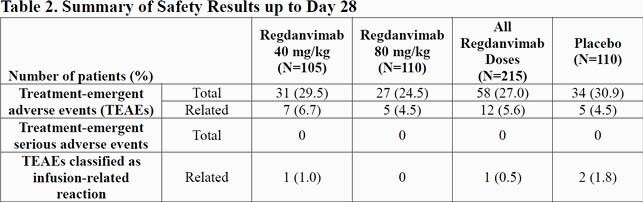

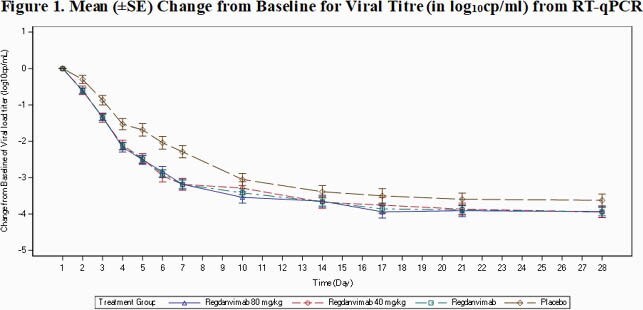

**Conclusion:**

Results from the first part of the study indicate that regdanvimab may lower the rate of hospitalisation or requirement of oxygen supplementation, with the greatest benefit noted in patients at high-risk of progressing to severe COVID-19. The second part of the study remains ongoing and blinded. Therefore, results for the primary endpoint are forthcoming and will be presented at IDWeek.

**Disclosures:**

**Michael G. Ison, MD, MS**, **Celltrion, Inc.** (Consultant) **Jin Yong Kim, MD, MPH**, **Celltrion, Inc.** (Scientific Research Study Investigator) **Oana Sandulescu, MD, PhD**, **Algernon Pharmaceuticals** (Scientific Research Study Investigator)**Atea Pharmaceuticals** (Scientific Research Study Investigator)**Celltrion, Inc.** (Scientific Research Study Investigator)**Diffusion Pharmaceuticals** (Scientific Research Study Investigator)**Regeneron Pharmaceuticals** (Scientific Research Study Investigator) **Liliana-Lucia Preotescu, MD, PhD**, **Celltrion, Inc.** (Scientific Research Study Investigator) **Norma Erendira Rivera Martinez, MD**, **Celltrion, Inc.** (Scientific Research Study Investigator) **Marta Dobryanska, MD**, **Celltrion, Inc.** (Scientific Research Study Investigator) **Victoria Birlutiu, Assoc. Prof. M.D. Ph.D.**, **Celltrion, Inc.** (Scientific Research Study Investigator)**Lucian Blaga University of Sibiu, Romania & Hasso Plattner Foundation** (Research Grant or Support) **Egidia Gabriela Miftode, MD, PhD**, **Celltrion, Inc.** (Scientific Research Study Investigator) **Natalia Gaibu, MD**, **Celltrion, Inc.** (Scientific Research Study Investigator) **Olga Adriana Caliman-Sturdza, MD, PhD**, **Celltrion, Inc.** (Scientific Research Study Investigator)**Stefan cel Mare University of Suceava, Romania** (Research Grant or Support) **Simin-Aysel Florescu, MD, PhD**, **Celltrion, Inc.** (Scientific Research Study Investigator) **Anca Streinu-Cercel, MD, PhD, Assoc.Prof. Infectious diseases**, **Algernon Pharmaceuticals** (Scientific Research Study Investigator)**Atea Pharmaceuticals** (Scientific Research Study Investigator)**Celltrion, Inc.** (Scientific Research Study Investigator)**Diffusion Pharmaceuticals** (Scientific Research Study Investigator)**Regeneron Pharmaceuticals** (Scientific Research Study Investigator) **Sang Joon Lee, n/a**, **Celltrion, Inc.** (Employee) **Sung Hyun Kim, n/a**, **Celltrion, Inc.** (Employee) **Il Sung Chang, n/a**, **Celltrion, Inc.** (Employee) **Yun Ju Bae, n/a**, **Celltrion, Inc.** (Employee) **Jee Hye Suh, n/a**, **Celltrion, Inc.** (Employee) **Mi Rim Kim, n/a**, **Celltrion, Inc.** (Employee) **Da Re Chung, n/a**, **Celltrion, Inc.** (Employee) **Sun Jung Kim, n/a**, **Celltrion, Inc.** (Employee) **Seul Gi Lee, n/a**, **Celltrion, Inc.** (Employee) **Ga Hee Park, n/a**, **Celltrion, Inc.** (Employee) **Joong Sik Eom, MD, PhD**, **Celltrion, Inc.** (Consultant)

